# Microstructures, Mechanical and Corrosion Properties of the Extruded AZ31-xCaO Alloys

**DOI:** 10.3390/ma11081467

**Published:** 2018-08-18

**Authors:** Yalin Lu, Yang Zhang, Mengqi Cong, Xingcheng Li, Wenting Xu, Leipeng Song

**Affiliations:** 1 School of Materials Engineering, Jiangsu University of Technology, Changzhou 213001, China; zhangyang@jsut.edu.cn (Y.Z.); xuwenting@jsut.edu.cn (W.X.); 2 Key Laboratory of Advanced Materials Design and Additive Manufacturing of Jiangsu Province, Changzhou 213001, China; congmq@jsut.edu.cn (M.C.); sgylxc@jsut.edu.cn (X.L.); songleipeng0130@163.com (L.S.)

**Keywords:** AZ31-xCaO alloy, microstructures, mechanical properties, corrosion properties

## Abstract

The effects of the extrusion process and CaO addition amount on microstructure, mechanical, and corrosion properties of AZ31 alloys were investigated by means of optical microscopy (OM), scanning electron microscopy (SEM), energy dispersive spectroscopy (EDS), standard tensile testing, and so on. The grain size of AZ31 or AZ31-1%CaO alloy becomes larger with increasing extrusion temperature. The grain size of AZ31-1%CaO alloy is much smaller than that of AZ31 alloy at the same extrusion temperature. In addition, the formation of the Al_2_Ca phase caused by CaO addition refines the grain size, and the recrystallization of AZ31-1%CaO alloy is improved significantly. The recrystallization grains distribute more uniformly as the increase of extrusion ratio, and the completely recrystallized grains distribute uniformly in the form of equiaxed crystals with an extrusion ratio of 9. Tensile testing results show that extruded AZ31-1%CaO alloy at the extrusion temperature of 300 °C and an extrusion ratio of 9 exhibits the best mechanical properties. While corrosion properties of AZ31 alloys decreases due to the addition of CaO.

## 1. Introduction

Mg alloys are widely applied in the fields of automotive and aerospace due to their specific strengths and low density. Up to now, Mg-Al alloy system is given to an attractive attention, such as Mg-Al-Zn (AZ) or Mg-Al-Mn (AM) alloys, while they are limited because of poor strength and corrosion resistance [1,2,3]. Besides that, rapid ignition that is caused by serious oxidation of magnesium at high temperature is also a big problem [4,5]. Thus some ignition-proof alloying elements, such as Be [6], Re [7,8], Ca [9], and so on, are added into Mg alloys, reducing the oxidation of magnesium melting. In addition, adding alloying elements can also reduce the use of SF_6_ and CO_2_ gas causing a global thermal effect. Because the alloying element is difficult to handle due to its high reactivity in atmospheric conditions, some oxides are developed for ignition-proof magnesium alloy [10,11].

Recently, cheaper CaO with good stability and easy achievement has been developed to prepare the oxidation- and ignition-proof Mg alloys [4,11,12,13,14,15]. Lee et al. [12] investigated microstructural evaluation of oxide layers in AM60 alloys with different CaO addition, and founded that CaO encouraged the columnar growth of a denser and thicker oxide layer in the AM60 alloy, which could effectively reduce the oxidation of Mg alloys. They [4] also pointed out that the ignition resistances of Mg-Al alloys under all conditions greatly increased by CaO addition, which could be contribute to the thin and dense oxide layer of MgO and CaO. Kwak et al. [13] also pointed out that a small amount of Ca (CaO) increased the ignition temperature of the Mg-9.5Zn-2.0Y alloy by about 90 K. In addition, Jang et al. [14] studied the damping capacity and mechanical properties of Mg alloy with CaO addition, and reported that CaO increased the certain mechanical properties of Mg. Choi et al. [15,16] studied the microstructures and mechanical properties of friction stir welded AZ31 or AZ91 alloys with CaO, they pointed out that an intermetallic compound of Al_2_Ca was formed, and hardness of the stir zone was harder than the base metal, which is likely due to the presence of fine grains and thermally stable intermetallic compounds. Jun et al. [17] studied the effects of 0.3% CaO on the microstructure and damping properties of AM50 casting alloys, and founded that the AM50-CaO alloy showed a higher damping level within the strain-amplitude dependent region than the AM50 alloy. Bae et al. [18] also investigated mechanical properties of AZ31 alloys with CaO processed by equal-channel angular pressing, and founded that the improved strength in the AZ31-CaO alloy was attributed to the formation of fine Al_2_Ca precipitates that experience breaking-up through ECAP and accelerate the microstructural refinement. At present, the mechanical and corrosion mechanism of Mg alloys with CaO remains unknown.

In the present study, a small amount of CaO was added to AZ31 Mg alloy, the effects of the extrusion process and CaO addition amount on microstructure, mechanical, and corrosion properties of AZ31 alloy are investigated, which provides a theoretical and experimental basis for the development of new magnesium alloys.

## 2. Materials and Methods

The base metal used in this study is commercial AZ31 Mg alloys. Table 1 shows its chemical composition. AZ31 Mg alloy was melted in a steel crucible while using an electric resistance furnace at 720 °C under a mixture gas of SF_6_ and CO_2_, and then CaO was added into the melting. Finally, the melt was pouring into a steel mold with diameter of 50 mm. In order to reduce and eliminate the composition segregation, AZ31-xCaO alloys were pre-heated at 400 ± 5 °C for 6 h in a resistance furnace.

The extrusion billets with diameter of 40 mm and length of 38 mm were conducted by vertical extruder with different extrusion ratios at different temperatures, and water quenched.

The specimen was cut to expose a surface on which observing microstructure along the extrusion direction. Specimens were mechanically polished, followed by chemical etching with an etching solution (1 mL nitric acid + 1 mL acetic acid + 1 g oxalic acid + 150 mL water). A MR5000 optical microscope (Zeiss, Primotech, Berlin, Germany) and ZEISS Gemini500 scanning electron microscope (FESEM, Carl Zeiss, Oberkochen, Germany) were used for observing the microstructure of specimens. In order to test the mechanical properties of samples, the tensile specimens were processed along the extrusion direction. Tensile testing was proceeded with a tensile strain rate of 2 mm/min at room temperature on the CMT-5205 universal testing machine (WANCE Co., Ltd., Shenzhen, China). The obtained tensile strength, yield strength, and elongation value were based on the average of three tests.

In order to understand the effect of CaO addition on corrosion of AZ31 alloy, potentiodynamic electrochemical tests were performed in a mini cell system (MCS): a working electrode in contact with the plastic tip and filled with 3.5 wt % NaCl solution; inside, a saturated calomel electrode (SCE) and a platinum plate were used as reference electrode and auxiliary electrode, respectively. The specimen was used for a potentiodynamic polarization test, which was measured at a scanning rate of 0.3 mV/s. EIS measurements were carried out using an electrochemical interface and a Solartron 1260 frequency response analyzer with a frequency range from 100,000 to 0.01 Hz. Surface product of the immersed samples was identified by scanning electronic microscopy (SEM).

## 3. Results and Discussion

### 3.1. Effect of CaO on Microstructure of AZ31 Alloy

**Figure 1:**
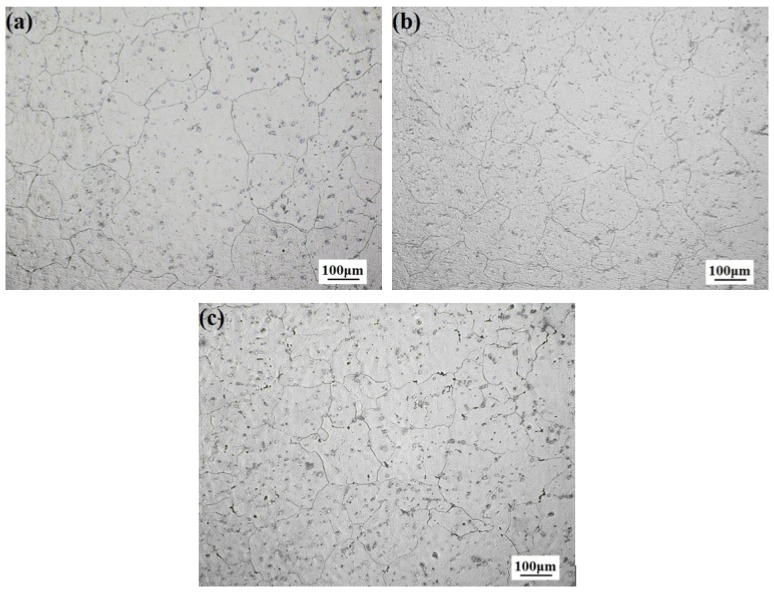
Optical microstructures of homogenization alloys. (**a**) AZ31; (**b**) AZ31-0.5%CaO; and, (**c**) AZ31-1%CaO.

Figure 1a–c shows the microstructure of AZ31 alloy with different CaO addition. It can be seen that, after homogenization heat treatment under 400 °C for 6 h, some phases are solid solution, grain boundary becomes clear, indicating that CaO has a refinement effect on AZ31 alloy. The grain size of AZ31 alloy is large, as shown in Figure 1a, AZ31-0.5%CaO alloy shown in Figure 1b exhibits smaller grain, while the smallest grain can be obtained after 1%CaO, as shown in Figure 1c. Therefore, the refinement effect is more evident with the increasing of CaO addition.

### 3.2. Effect of Extrusion on Microstructure and Properties of AZ31 Alloy

#### 3.2.1. Effect of Extrusion Temperature on Microstructure of AZ31-xCaO Alloy

**Figure 2:**
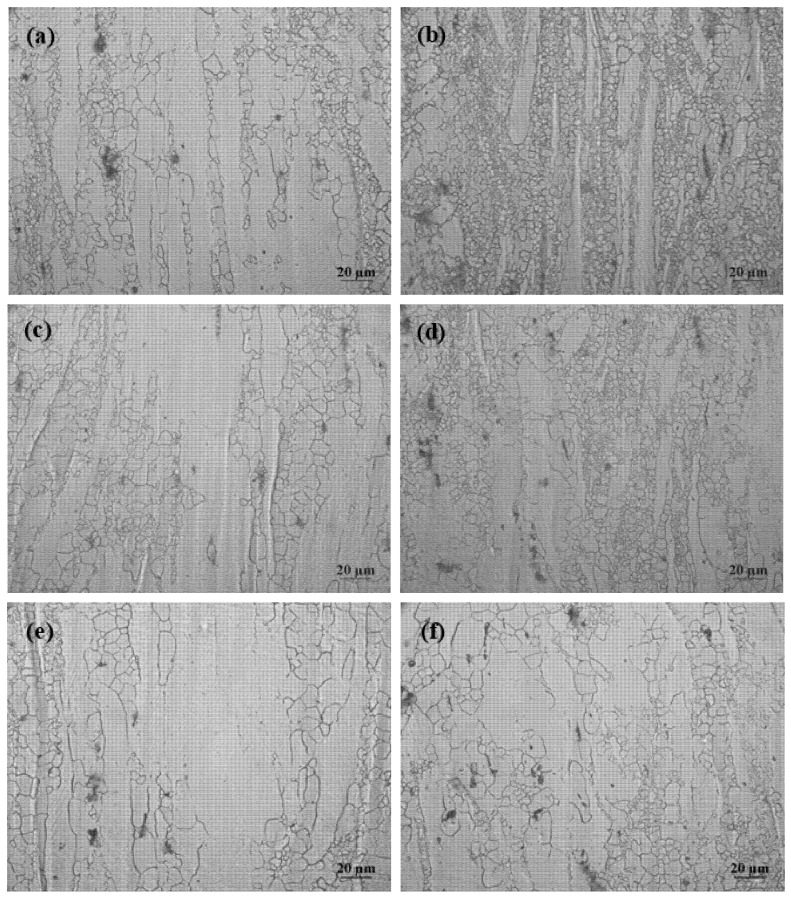
Microstructures of AZ31 alloy (**a**,**c**,**e**) and AZ31-1%CaO alloy (**b**,**d**,**f**) at a extrusion ratio of 6 under different extrusion temperatures: (**a**,**b**) 300 °C; (**c**,**d**) 350 °C; and, (**e**,**f**) 400 °C.

Figure 2 shows microstructures of AZ31 and AZ31-1%CaO alloys under different extrusion temperatures. It can be found that the extrusion alloys mainly consist of α-Mg and the second phases distributed in grain and grain boundary. The grain size of AZ31 or AZ31-1%CaO alloy becomes larger with an increasing extrusion temperature, as shown in Figure 2a,c,e or Figure 2b,d,f, indicating that the grain size of AZ31 alloy still becomes larger although adding 1%CaO. The grain size of AZ31-1%CaO alloy is much smaller than that of AZ31 alloy at the same extrusion temperature. In addition, the formation of Al_2_Ca phase that is caused by CaO addition refines the grain size, and the recrystallization of AZ31-1%CaO alloy is improved significantly [19].

(1) $$Z=\overset{•}{\varepsilon }\exp\left|\frac{Q}{RT}\right|$$

(2) lnD=klnZ+b

where *Q* is activation energy; $\overset{•}{\varepsilon }$ is strain rate; *R* is molar gas constant; *D* is the dynamic recrystallization size; and, *K* and *B* are constant.

The Formulas (1) and (2) show that the strain rate is more slowly affected by the temperature when the extrusion temperature is certain. As a certain strain rate, the higher the temperature, the smaller the z parameter, and the larger the dynamic recrystallization size.

In the process of extrusion, the sub-crystalline structure is firstly formed at the grain boundary under the effect of extrusion stress and extrusion heat. Then, fine large angle grains are formed through the mechanism of sub-crystal consolidation. These grain boundaries are further merged and rotated through the migration and absorption of dislocation, further sub-grains combine with each other and rotate, and finally finer dynamic recrystallized grains are formed. The recrystallized grains absorb more heat and promote grain growth due to the increase of extrusion temperature [20,21,22].

#### 3.2.2. Effect of Extrusion Ratio on Microstructure of AZ31-xCaO Alloy

**Figure 3:**
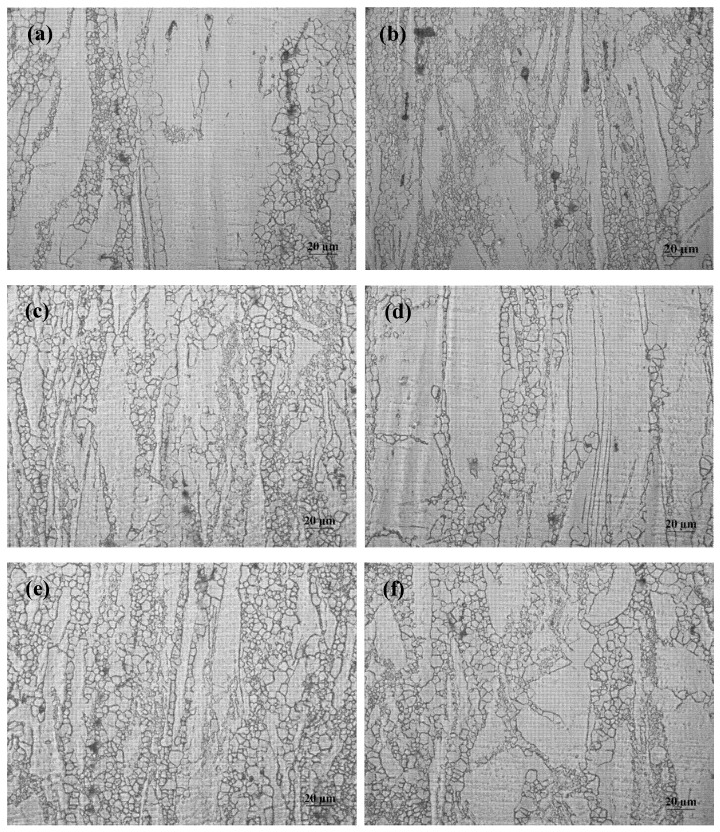
Microstructures of AZ31 (**a**,**c**,**e**) and AZ31-1%CaO alloy (**b**,**d**,**f**) at different extrusion ratios under 300 °C. (**a**,**b**) extrusion ratio is 3; (**c**,**d**) extrusion ratio is 6; and, (**e**,**f**) extrusion ratio is 9.

Microstructures of AZ31 and AZ31-1%CaO alloys under different extrusion ratios are displayed in Figure 3. It can be seen that AZ31 alloy structure mainly consists of α-Mg and the second phase distributed in the matrix. Meanwhile, the lathy fibrous microstructure also can be clearly observed. When the extrusion ratio is 3 (shown in Figure 3a,b), the recrystallized grains are formed in the matrix, coarse grains are broken, and lead to the formation of fine microstructure around adjacent coarse grains under the extrusion stress. In addition, the degree of crystallization increases with increasing extrusion ratio. When the extrusion ratio is 9, the recrystallization degree is the highest, and the grain distribution is the most uniform. It also can be found that the microstructure evolution of AZ31-1%CaO alloy is similar to that of the AZ31 alloy. As an extrusion ratio is 6 (shown in Figure 3d,e), the recrystallization can reaches a relatively high level, the number and size of lathy fibrous structures decrease. When extrusion ratio reaches 9, the completely recrystallized grains distribute uniformly in the form of equiaxed crystals, as shown in Figure 3f.

The grain refinement and increase of recrystallization degree can be attributed to two aspects. On the one hand, increasing of extrusion ratio results in an increase of strain rate, thus the severe plastic deformation causes serious distortion of crystal structure, which can provide a powerful condition for dynamic recrystallization. On the other hand, a certain increasing temperature improves the nucleation rate and promotes recrystallization. Larger extrusion ratio produces more heat during the extrusion process, so the recrystallization degree is improved. In addition, the dynamic recrystallization can be repeated under certain conditions, and the grain size becomes smaller than the larger extrusion ratio.

#### 3.2.3. Orientations of Extruded AZ31-xCaO Alloy

**Figure 4:**
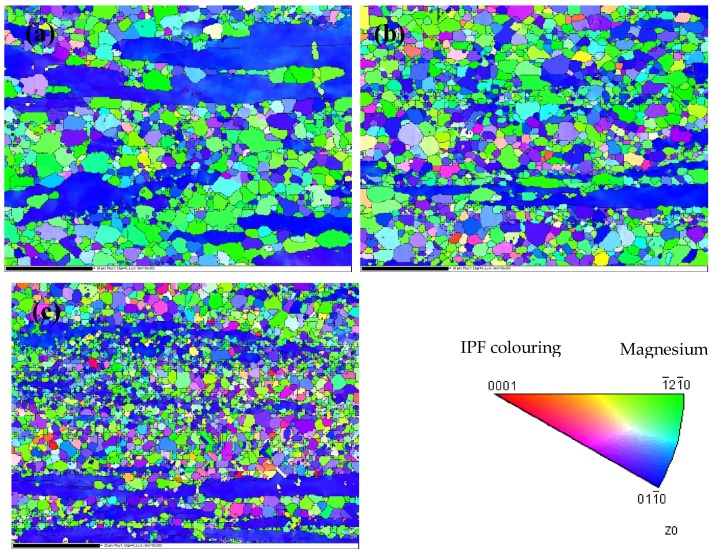
Orientation of the extruded AZ31-xCaO alloys. (**a**) AZ31; (**b**) AZ31-0.5%CaO; and, (**c**) AZ31-1%CaO.

Orientations of extruded AZ31-xCaO alloys are shown in Figure 4. The apparent recrystallization of extruded alloys at different temperatures also can be observed clearly. The microstructure is composed of non-recrystallized fibers and equiaxed grains. According to the analysis of EBSD results, the average grain size of AZ31 is 1.95 μm and the maximum grain size is 47.5 μm. It can be observed that the recrystallization degree of AZ31-0.5%CaO alloy is improved, which is shown in Figure 4b. Besides, the average grain size is 1.79 μm, and the maximum grain size is 23.77 μm. Figure 4c is an orientation image of AZ31-1%CaO with an average grain size of 1.39 μm and a maximum grain size of 20.73 μm. The grain number of extruded alloys in the same area is 1023, 1507, and 2325, respectively. The average grain size and grain number all confirm that more CaO content promotes recrystallization more obviously.

**Figure 5:**
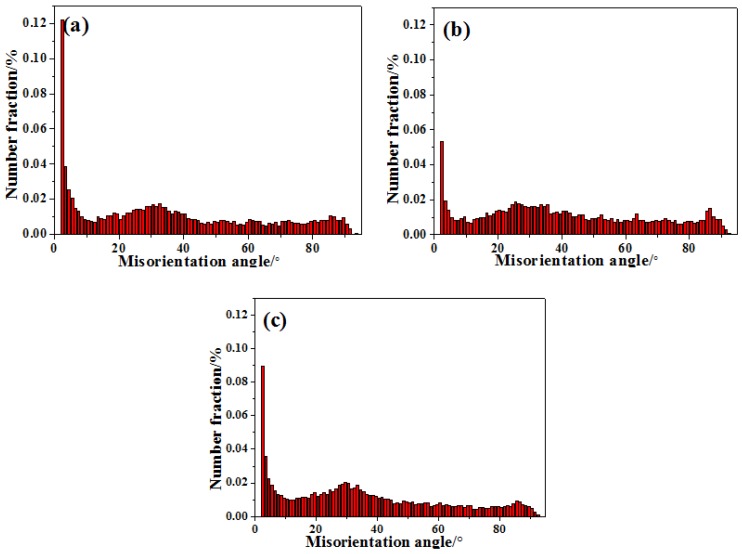
Disorientation map of grain boundary in AZ31-xCaO alloys. (**a**) AZ31; (**b**) AZ31-0.5%CaO; and, (**c**) AZ31-1%CaO.

Figure 5 shows disorientation differences of grain boundary in AZ31-xCaO alloys. By statistics, the fraction of small angle grain boundary in extruded AZ31 alloy shown in Figure 5a is 25.4%, and that in AZ31-0.5%CaO alloy is 13.2%, indicating that CaO can promote the dynamic recrystallization during the extrusion process. As shown in Figure 5c, the proportion of small angle grain boundary in AZ31-1%CaO alloy (22.8%) decreases slightly, which also confirms that CaO can promote recrystallization. In addition, the ratio peak in 30 ± 5° and 86 ± 5° is corresponding to {0001} base plane and {10$\overset{-}{1}$2} twins, respectively.

**Figure 6:**
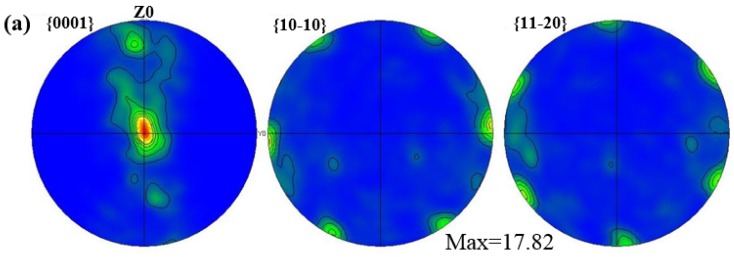

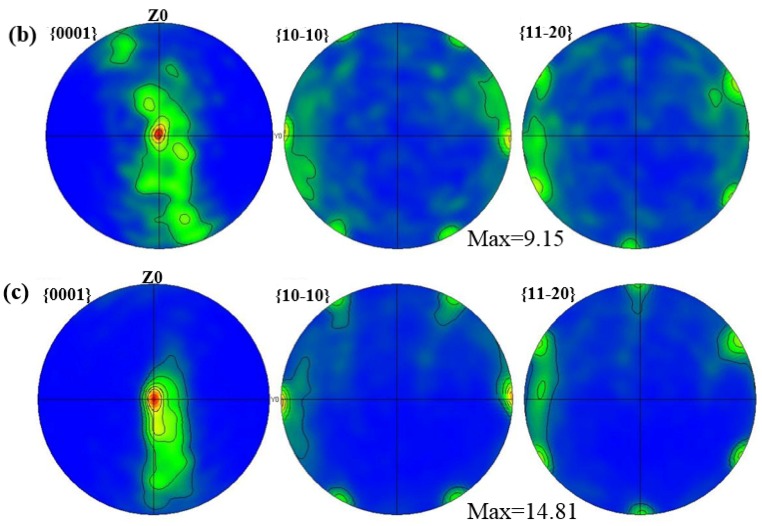
The texture strength of extruded AZ31-xCaO alloys. (**a**) AZ31; (**b**) AZ31-0.5%CaO; and, (**c**) AZ31-1%CaO.

Figure 6a–c displays the texture strength of extruded AZ31-xCaO alloys. It can be seen that CaO addition is beneficial to improve the texture strength of the AZ31 alloy. The texture strength of AZ31 alloy is 17, while that of alloy with 0.5%CaO decreases by 52.9%. That in AZ31-1%CaO decreased by 23.5%. Therefore, the CaO addition can improve the texture strength of AZ31 alloy significantly.

#### 3.2.4. Effect of Extrusion on Mechanical Properties of AZ31-xCaO Alloy

**Figure 7:**
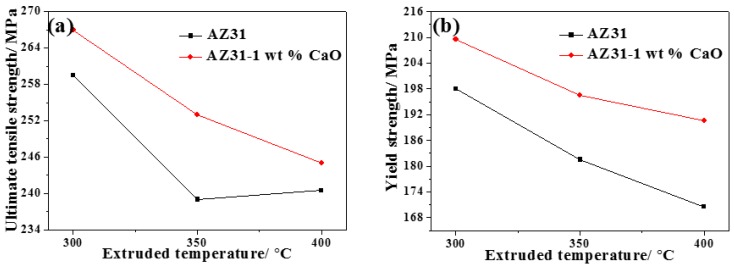

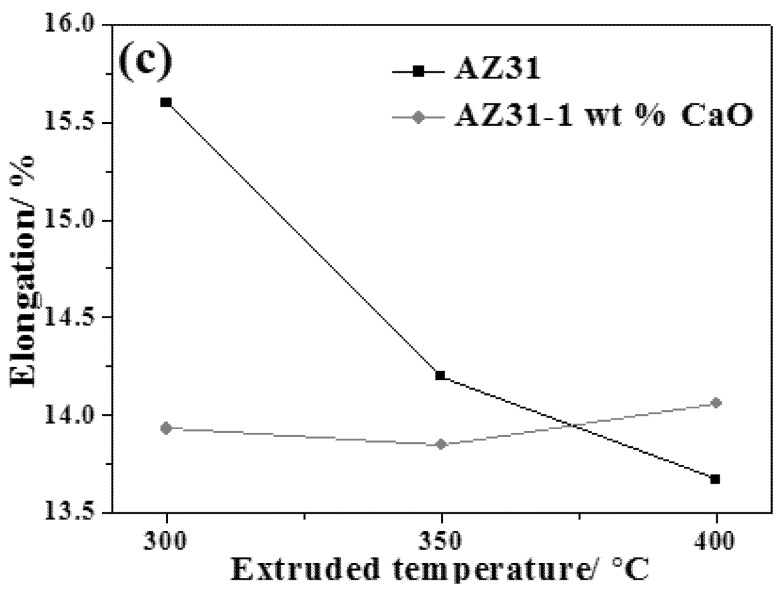
Tensile properties of AZ31 and AZ31-1%CaO alloys under different extrusion temperatures. (**a**) Ultimate tensile strength; (**b**) yield strength; and, (**c**) elongation.

Mechanical properties of AZ31 and AZ31-1%CaO alloys under different extrusion temperatures are shown in Figure 7. It can be seen that yield strength and elongation of extruded AZ31 alloy at room temperature decrease with an increasing of extrusion temperature, while ultimate tensile strength decreases firstly and then increases. Ultimate tensile strength and yield strength of extruded AZ31 alloy with 1%CaO decrease with increasing temperature, while the elongation of the alloy (shown in Figure 7c) decreases firstly and then increases. In addition, ultimate tensile strength and yield strength of the alloy with 1%CaO are higher than those of AZ31 alloy at the same extrusion temperature, while elongation is lower than that of AZ31 alloy at the extrusion temperature of 300 °C and 350 °C. When the extrusion temperature is 300 °C, ultimate tensile strength of AZ31 alloy is 259.5 MPa, its yield strength is 198 MPa, and elongation is 15.6%, while the tensile strength of AZ31-1%CaO alloy (267 MPa) increases by 2.9% than that of AZ31 alloy, its yield strength (209.5 MPa) increases by 5.8%, and elongation is 13.93%. Therefore, AZ31-1%CaO alloy exhibits the best mechanical properties.

Generally, the microstructure of alloys affects observably mechanical properties. With increasing the extrusion temperature, grain after the crystallization will grow up because of absorbing more heat to make the grains grow up. The higher the extrusion temperature, the more heat grains can absorb, thus the grain size will be larger [23,24]. The hindrance of dislocation and the restraint during tensile deformation will become weaker due to less grain boundary, so the yield strength and hardening effect of the alloy will be reduced.

Generally, elongation is inversely proportional to the grain size, according to the Hall-petch formula. In this paper, large grain that is caused by increasing extrusion temperature will decrease the elongation of AZ31-xCaO alloys.

**Figure 8:**
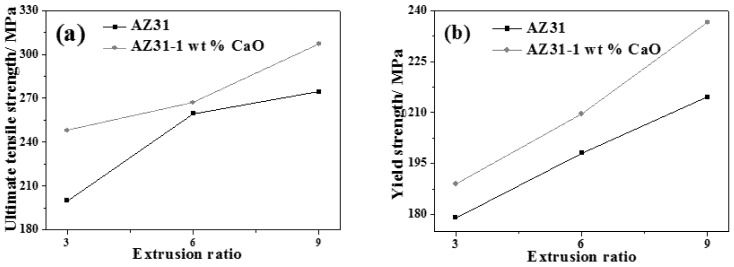

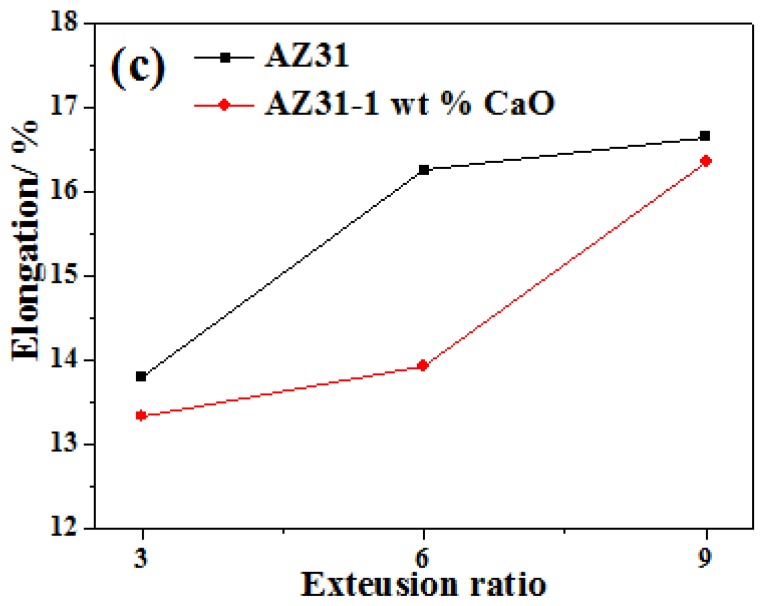
Tensile properties of AZ31 and AZ31-1%CaO alloys at different extrusion ratios. (**a**) Ultimate tensile strength; (**b**) yield strength; and, (**c**) elongation.

Mechanical properties of AZ31 and AZ31-1%CaO alloys at different extrusion ratios are shown in Figure 8. It can be seen that ultimate tensile strength, yield strength, and elongation of AZ31-xCaO alloys all increase with the increase of extrusion ratio. When the extrusion ratio is 9, the mechanical properties of AZ31-xCaO alloys is the best. In addition, ultimate tensile strength and yield strength of the alloy with 1%CaO are higher than those of AZ31 alloy at the same extrusion ratio, while elongation is lower than that of AZ31 alloy.

The change of extrusion ratio directly affects strain rate, which can be calculated by the following formula:

(3) $$\overset{•}{\varepsilon }EX=\frac{6vd_{0}^{2}\ln{\left(d_{0}/d_{m}\right)}^{2}\tan\phi }{d_{0}^{3}-d_{m}^{3}}$$

where *v* is the compression speed of extrusion rod (mm/min), *φ* is the half angle of concave die, *d*_0_ is the diameter of blank, and *d_m_* is the diameter of extrusion bar.

From the above formula, the smaller diameter of extrusion bar will lead to a smaller strain rate in the same extrusion speed and billet size. The influence of extrusion on microstructures and properties is mainly related to the deformation and stress state of alloys. With the increase of extrusion ratio, as seen in Figure 3, Figure 4, Figure 5 and Figure 6, the deformation of extruded alloys increases, the finer extrusion streamlines and more uniformly distributed grains with smaller size. Therefore, the tensile strength and elongation of extruded alloys are improved significantly. In addition, non-recrystallization fibrous may cause the processing hardening in some extent, resulting in the improvement of yield strength.

#### 3.2.5. Corrosion Properties of AZ31-xCaO Alloys

**Figure 9:**
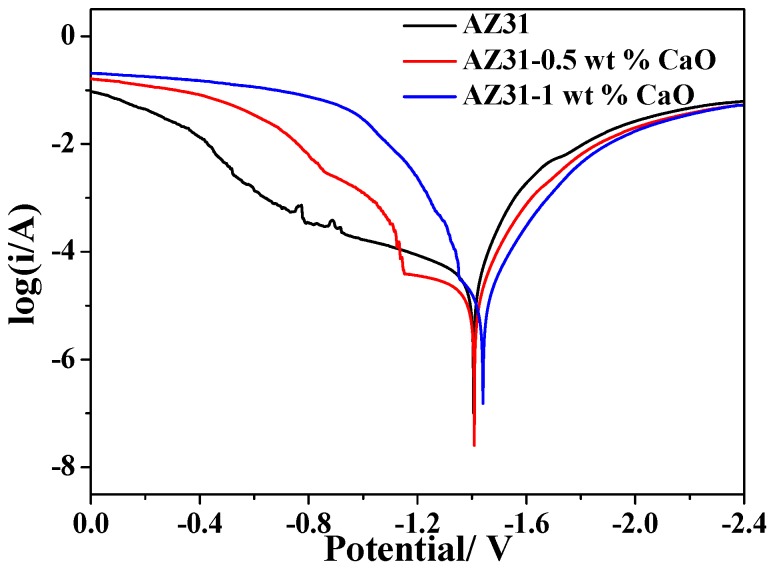
The polarization curves of AZ31-xCaO alloys.

The polarization curves of AZ31-xCaO alloys are displayed in Figure 9. Meanwhile, self-corrosion potential of extruded alloys shown in Table 2, which can be calculated from Figure 9. It can be seen that corrosion potential of extended alloys becomes smaller with increasing CaO addition, and it shifts to negative direction. The self-corrosion potential of AZ31 alloy is −1.406 V, that of AZ31-0.5%CaO alloy is −1.409 V, which is smaller than that of AZ31 alloy. As the amount of CaO addition is 1%, the self-corrosion potential of the alloy is −1.441 V, which reduces by 35 mV. It can be concluded that corrosion resistance of extruded alloys decreases with increasing amount of CaO addition.

In general, the cathode polarization curve shows the process of hydrogen production though the reaction of extruded alloys with water, and the anodic polarization curve is expressed as the dissolving process of magnesium, i.e.,

2H_2_O + 2e^−^ = H_2_ + 2OH^−^ (cathodic reaction)

Mg = Mg^2+^ + 2e^−^ (anodic reaction)

Mg^2+^ + 2OH^−^ = Mg(OH)_2_ (formation of corrosion products)

**Figure 10:**
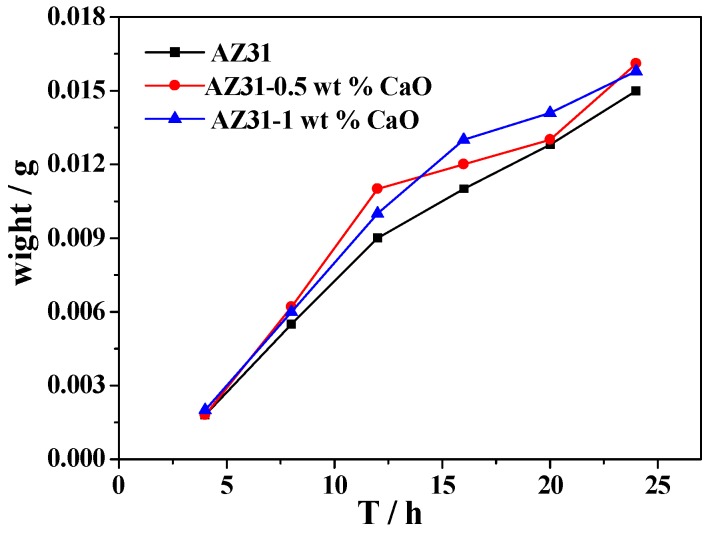
Electrochemical impedance spectra of extruded AZ31-xCaO alloys.

Electrochemical impedance spectra of extruded AZ31-xCaO alloys are shown in Figure 10. The capacity-resistance arc appears in the high-frequency and intermediate frequency regions for AZ31 alloy, and in the low-frequency region for the alloys with CaO addition. Generally, high-frequency capacity-resistance arc is caused by the change of charge transfer resistance of the material surface, and low-frequency capacity arc induced by the magnesium ions diffusion of Mg(OH)_2_ corrosion products, while intermediate frequency inductance arc is caused by the adsorption of magnesium ions on the specimen surface without passivation film. The larger the radius of tolerance arc, the greater the surface resistance of alloys, and the better the corrosion resistance of alloys. These are consistent with the results of polarization curve and self-corrosion potential.

**Figure 11:**
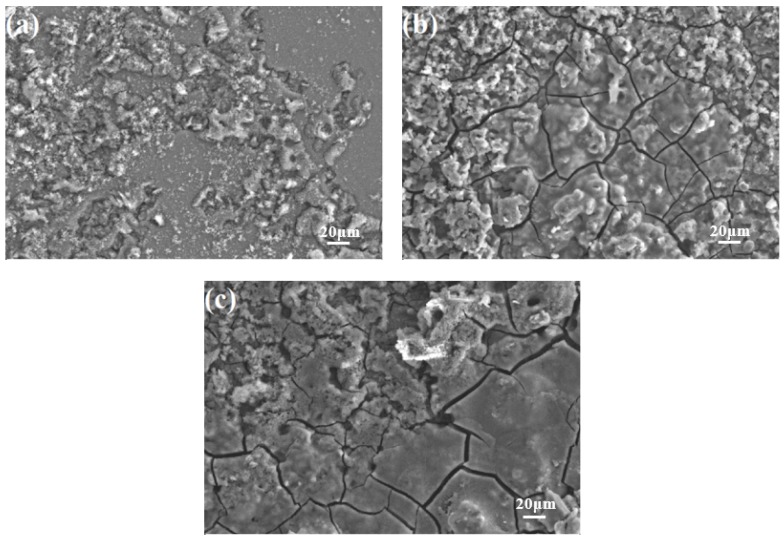
The corrosion morphologies of AZ31-xCaO alloys. (**a**) AZ31; (**b**) AZ31-0.5%CaO; and, (**c**) AZ31-1%CaO.

Figure 11 displays the corrosion morphologies of AZ31-xCaO alloys after immersion in 3.5%NaCl solution. EDS patterns of white regions are mainly Mg, O, and Al elements, indicating that the corrosion products are mainly Mg or Al hydroxides. Corrosion of alloys become serious with an increasing CaO amount, as shown in Figure 11. The surface adhesion corrosion products of AZ31 alloy are least, indicating that AZ31 alloy exhibits a mild corrosion. Some cracks appear on the surface of AZ31-0.5%CaO alloy, and the number of cracks increases with further increasing CaO. Besides, there are obvious corrosion pits on the surface of AZ31-1%CaO alloy, indicating the occurrence of pitting.

**Figure 12:**
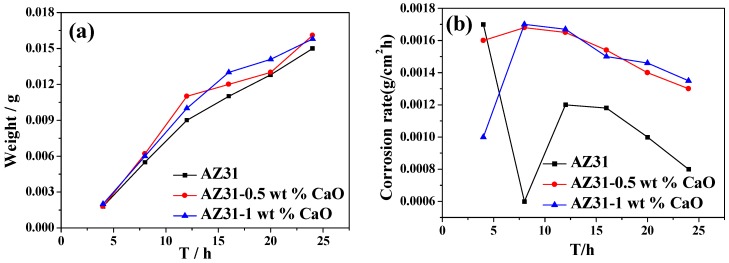
The corrosion weight loss curve (**a**) and corrosion rate curve (**b**) of AZ31-xCaO alloys.

Figure 12 is the corrosion weight loss and corrosion rate curves of extruded AZ31-xCaO alloys after immersion in 3.5% NaCl solution. It can be found that corrosion degree of extruded alloys increase over time, but their corrosion rates decrease gradually. In addition, the weight loss rate of extruded alloy with CaO is significantly higher than that of AZ31 alloy, which shows that adding CaO reduces the corrosion resistance of AZ31 alloy.

Al_2_Ca formed by in-situ reaction of CaO with Al acts as cathode, and it causes corrosion micro-cell with α-Mg matrix, thus electrode reaction is formed, and it promotes the dissolution of α-Mg matrix [5,25]. Meanwhile, CaO addition leads to the activation of Mg anode. All of these result in the destruction and fall off of α-Mg matrix during the electrode reaction, and it forms a pitting corrosion. Ca dissolved in NaCl solution will be reduced by Mg and deposits back to the surface of alloy due to its higher activity, which breaks the integrity of passivation film, and also impedes the adhesion of corrosion products, thus the surface of alloy is directly exposed to solution, further aggravating the corrosion process. Therefore, corrosion products become more and more loose and prone to fall off. In addition, increasing Al_2_Ca causes more micro-batteries due to the increase of CaO addition; α-Mg matrix dissolves faster, thus reducing the corrosion resistance of AZ31 alloys.

## 4. Conclusions

Effects of extrusion process and CaO addition on the microstructure, mechanical, and corrosion properties of extruded AZ31 alloy have been investigated in the present work and the main conclusions can be summarized, as follows:

(1) The grain size of AZ31 or AZ31-1%CaO alloy becomes larger with increasing extrusion temperature. The grain size of AZ31-1%CaO alloy exhibits much smaller than that of AZ31 alloy at the same extrusion temperature. The recrystallization grains distribute more uniformly as the increase of extrusion ratio, and the completely recrystallized grains distribute uniformly in the form of equiaxed crystals as an extrusion ratio of 9.
(2) The yield strength and elongation of extruded AZ31 alloy at room temperature decrease with increasing of extrusion temperature, while ultimate tensile strength decreases firstly and then increases. The ultimate tensile strength, yield strength, and elongation of AZ31-xCaO alloys all increase with the increase of extrusion ratio. The extruded AZ31-1%CaO alloy at the extrusion temperature of 300 °C and an extrusion ratio of 9 exhibits the best mechanical properties.
(3) Corrosion potential of extended alloys becomes smaller with increasing CaO addition, and shifts to negative direction. Corrosion degree of extruded alloys increase over time, while their corrosion rates decrease gradually. The weight loss rate of extruded alloy with CaO is significantly higher than that of AZ31 alloy. Corrosion resistance of extruded alloys decreases with increasing CaO addition, which can be attributed to the addition of CaO.

## Figures and Tables

**Table 1:** The chemical composition of commercial AZ31 Mg alloys (%).

Al	Zn	Mn	Fe	Cu	Ni	Si	Mg
2.5-3.5	0.6-1.4	0.2-1.0	0.003	0.01	0.001	0.08	Bal.

**Table 2:** Self-corrosion potential of extruded alloys.

Extruded Alloys	Self-Corrosion Potential E_corr_/V
AZ31	−1.406
AZ31-0.5%CaO	−1.409
AZ31-1%CaO	−1.441
